# Comprehensive identification of essential pathways and transcription factors related to epilepsy by gene set enrichment analysis on microarray datasets

**DOI:** 10.3892/ijmm.2014.1843

**Published:** 2014-07-09

**Authors:** KAN HE, WEIZHONG XIAO, WENWEN LV

**Affiliations:** 1 Center for Stem Cell and Translational Medicine, School of Life Sciences, Anhui University, Hefei, Anhui 230601, P.R. China; 2 Department of Neurology, Shanghai Pudong Hospital, Fudan University Pudong Medical Center, Pudong, Shanghai 201399, P.R. China

**Keywords:** epilepsy, pathway, gene set enrichment analysis, peroxisome proliferator-activated receptor

## Abstract

Epilepsy is a common chronic neurological disorder characterized by seizures or convulsions, and is known to affect patients with primary brain tumors. The etiology of epilepsy is superficially thought to be multifactorial; however, the genetic factors which may be involved in the pathogenesis of seizures have not yet been elucidated, particularly at the pathway level. In the present study, in order to systematically investigate the gene regulatory networks involved in epilepsy, we employed a microarray dataset from the public database library of Gene Expression Omnibus (GEO) associated with tumor-induced epileptogenesis and applied gene set enrichment analysis (GSEA) on these data sets and performed candidate transcription factor (TF) selection. As a result, 68 upregulated pathways, including the extracellular matrix (ECM)-receptor interaction (P=0.004) and peroxisome proliferator-activated receptor (PPAR) signaling pathways (P=0.045), as well as 4 downregulated pathways, including the GnRH signaling pathway (P=0.029) and gap junction (P=0.034) were identified as epileptogenesis-related pathways. The majority of these pathways identified have been previously reported and our results were in accordance with those reports. However, some of these pathways identified were novel. Finally, co-expression networks of the related pathways were constructed with the significant core genes and TFs, such as PPAR-γ and phosphatidylethanolamine-binding protein. The results of our study may contribute to the improved understanding of the molecular mechanisms of epileptogenesis on a genome-wide level.

## Introduction

Epilepsy is a common chronic neurological disorder characterized by epileptic seizures, which vary in their duration. These episodes can range from brief and nearly undetectable seizures to long periods of vigorous shaking (1,2). In epilepsy, seizures tend to recur, and have no immediate underlying cause, while seizures that occur due to a specific cause are not deemed to represent epilepsy (3). In the majority of cases, the cause of epilepsy is unknown, although some individuals develop epilepsy as a result of brain injury, stroke, brain cancer or drug and alcohol misuse, among others. Epileptic seizures are the result of excessive and abnormal cortical nerve cell activity in the brain (3).

Genetics are believed to be involved in the majority of cases of epilepsy, either directly or indirectly. Although some of the genes involved affect ion channels, other molecules such as enzymes, gamma-aminobutyric acid (GABA) and G protein-coupled receptors have been identified as single genes in which defects cause epilepsy; however, epilepsy may occur due to the interaction of multiple genes and environmental factors (4). The etiology of epilepsy is superficially thought to be multifactorial; however, the genetic factors that may be involved in the pathogenesis of seizures have not yet been elucidated. To study the gene regulatory networks involved in epilepsy, a variety of genome-wide studies have been performed by different groups using various systems and array platforms. The accumulated functional genomic data are freely available in the database of Gene Expression Omnibus (GEO, http://www.ncbi.nlm.nih.gov/geo/) (5,6), which provides a golden opportunity for compiling a comprehensive list of genetic factors underlying the etiology of epilepsy. According to the approach of differentially expressed gene analysis (DEGA) for studying gene expression profiles, hundreds of significant genes have been identified to be associated with epilepsy. However, few studies have focused on the associated pathways and transcription factors (TFs), as well as on the co-expression patterns at the multiple pathways level.

In the present study, we employed a microarray dataset of genome-wide gene expression profiling from GEO, which is associated with tumor-induced epileptogenesis. The most well-known method of gene set enrichment analysis (GSEA) was used to analyze the genomic data in order to uncover the regulatory mechanisms of human epilepsy caused by brain tumors at the multiple pathways level. GSEA is widely used to analyze gene expression profiles, particularly to identify pre-defined gene sets which exhibit significant differences in expression between samples from the control and treatment groups (7–9). The goal of GSEA is to determine other interesting categories (pathways) where the constituent genes show coordinated changes in expression over the experimental conditions, other than in the form of sets of differentially expressed genes (DEGs). One of the advantages of GSEA is the ability to highlight genes weakly connected to the phenotype through pathway analysis, which may be difficult to detect by using classical univariate statistics (7).

## Materials and methods

### Microarray data collection and pre-processing

We searched the GEO database (www.ncbi.nlm.nih.gov/geo/) for gene expression profiling studies related to epilepsy. Data were included in our re-analysis if they met the following criteria: i) the data were genome-wide; ii) the comparison was conducted between samples with epilepsy and controls; iii) complete microarray raw or normalized data were available. Finally, we selected the dataset of GSE32534 for our re-analysis, which was contributed by Niesen *et al* (10). In this dataset, genome-wide gene expression profiling was conducted using the Affymetrix Human Genome U133 Plus 2.0 Array and the RNA was derived from formalin-fixed paraffin-embedded (FFPE) peritumoral cortex tissue slides from 5-paired (seizure vs. non-seizure) low grade brain tumor patients. There were 5 biological replicates for epilepsy [samples from GSM805925 to GSM805929, marked with epilepsy (EP)-1, EP-2, EP-3, EP-4 and EP-5, respectively] and 5 for the controls [samples from GSM805930 to GSM805934, marked with control (CT)-1, CT-2, CT-3, CT-4 and CT-5, respectively].

For the assessment of the influence of pre-processing on the comparison, data pre-processing was performed using software packages developed in version 2.6.0 of Bioconductor and R version 2.10.1. Each Affymetrix dataset was background adjusted, normalized and log2 probe-set intensities were calculated using the Robust Multichip Averaging (RMA) algorithm in Affy package, as previously described (11).

### GSEA

Our GSEA of pathways and genes was performed using the Category package in version 2.6.0 of Bioconductor, as previously described (12). The goal of GSEA is to determine whether the members of a gene set ‘S’ are randomly distributed throughout the entire reference gene list ‘L’ or are primarily found at the top or bottom of the list. One of the advantages of GSEA is the relative robustness to noise and outliers in the data. In our analysis, the gene sets represented by <10 genes were excluded. The t-statistic mean of the genes was computed in each Kyoto Encyclopedia of Genes and Genomes (KEGG) pathway. Using a permutation test 1,000 times, the cut-off of the significance level P-values was selected as 0.05 for the significant pathways related to epilepsy. Accordingly, the significant pathways and genes were then identified under the comparison between the samples with epilepsy and no epilepsy. The following classification of identified pathways was based on the KEGG pathway maps br08901 of BRITE Functional Hierarchies in the KEGG database (http://www.genome.jp/kegg-bin/get_htext?br08901.keg). The annotation of significant genes in each pathway was performed by using biomaRt package, BioMart v. 0.8 rc3 (version of 0.8 release candidate 3; http://www.biomart.org/). Subsequently, clustering of the groups and genes was performed based on the identified gene expression in each significant pathway using the method of hierarchical clustering with Pearson correlation coefficient.

### Regulatory elements (REs) and TFs of co-regulated genes

We further employed a web server termed DiRE (Distant RE of co-expressed genes, http://dire.dcode.org/), based on the enhancer identification (EI) method, to predict common REs for our input genes which have co-function in each identified significantly related pathway (13). It predicts function-specific REs consisting of clusters of specifically-associated transcription factor binding sites (TFBSs), and it also scores the association of individual TFs with the biological function shared by the group of input genes. We selected a random set of 5,000 genes in the human genome (hg18) as the source of background genes. As a result, there are 2 major parameters of our predicted TFs, including TF occurrence which denotes the percentage of candidate RE containing a conserved binding site for a particular TF; and TF importance which denotes the product of TF occurrence and TF weight. As our candidate associated TFs with input gene sets, we selected the cut-off value of TF importance as >0.05.

## Results and Discussion

### Identification of significant pathways associated with epilepsy

In the study of Niesen *et al* (10), a number of DEGs between the 2 groups (epilepsy and the control) were identified using both the parametric unpaired Student’s t-test (345 probe sets representing 296 genes with fold-changes ≥2 plus P≤0.05) and the non-parametric rank product [377 probe sets representing 344 genes with a false discovery rate (FDR) of ≤0.3]. Seven DEGs, i.e., *C1QB*, *CALCRL*, *CCR1*, *KAL1*, *SLC1A2*, *SSTR1* and *TYRO3* were validated by qRT-PCR. Moreover, the pathway analysis using DAVID bioinformatics resources revealed that these DEGs were mainly enriched in focal adhesion, extracellular matrix (ECM)-receptor interaction and cell adhesion molecule (CAM) pathways.

Compared to the approach of DEGs, the strategy of GSEA we used in this study is likely to be more powerful than conventional single-gene methods in the study of complex diseases in which many genes make subtle contributions. According to our GSEA on the dataset of 10 samples by the comparison of epilepsy to the controls, in total, there were 72 significant pathways associated with epilepsy, whose P-values were <0.05, including 4 downregulated and 68 upregulated pathways. The 3 main pathways identified by Niesen *et al* (10) were also included in our results. Moreover, based on the KEGG pathway maps in the database of KEGG (http://www.genome.jp/kegg/), these 72 significant pathways could be mainly mapped into 6 functional classes, including cellular processes, environmental information processing, genetic information processing, human diseases, as well as metabolism and organismal systems. The details of the involved pathways in each class are presented in Tables I–V. The details of the associated genes in each significant pathway are available upon request (data not shown), including the information on probe set id and gene symbol.

**Figure 1:**
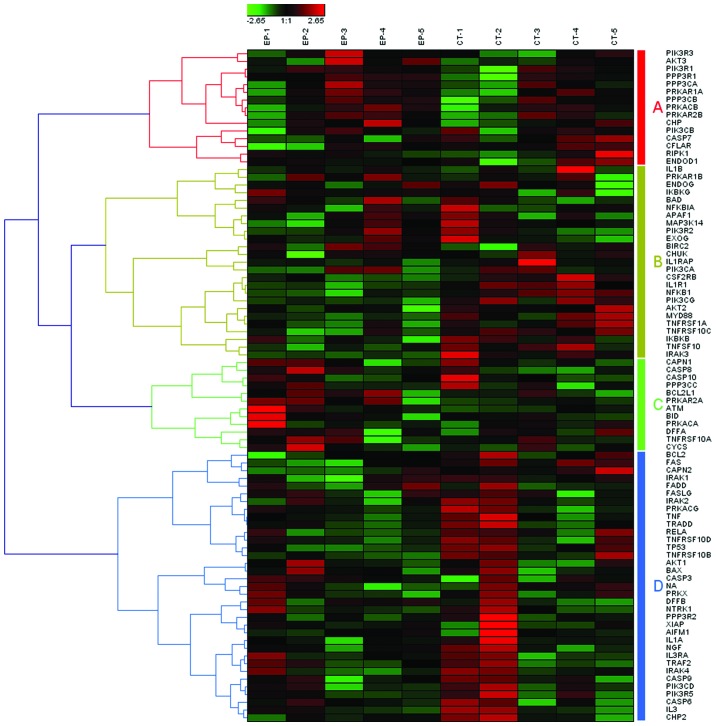
Hierarchical clustering of genes involved in the pathway of apoptosis related to epilepsy. In total, there were 87 genes involved in the pathway of apoptosis related to epilepsy, which may be clustered into 4 gene set groups (groups A–D) based on hierarchical clustering with Pearson correlation coefficient. Heatmap of 87 genes involved in the pathway of apoptosis in 5 epilepsy samples (from EP-1 to EP-5) and 5 control samples (non-epilepsy, from CT-1 to CT-5) is shown. EP, epilepsy; CT, control.

In the functional class of cellular processes, there was 1 significantly downregulated and 8 significantly upregulated pathways associated with epilepsy (Table I). These pathways were involved in cell communication, cell growth and death, cell motility, as well as transport and catabolism. Among these, apoptosis was one of the most significant pathways (P<0.001), which was classified into the functional group of cell growth and death. As is known, high-frequency stimulation (HFS) of the hippocampus may be a promising method in the treatment of epilepsy. It has been indicated that hippocampal HFS can protect hippocampal neurons against kainic acid (KA) neurotoxicity, and that the neuroprotective effects of HFS may be mediated through the inhibition of apoptosis (14). In total, there were 87 involved genes in the pathway of apoptosis related to epilepsy, which may be clustered into 4 gene set groups based on hierarchical clustering with Pearson correlation coefficient (Fig. 1, groups A–D).

**Figure 2:**
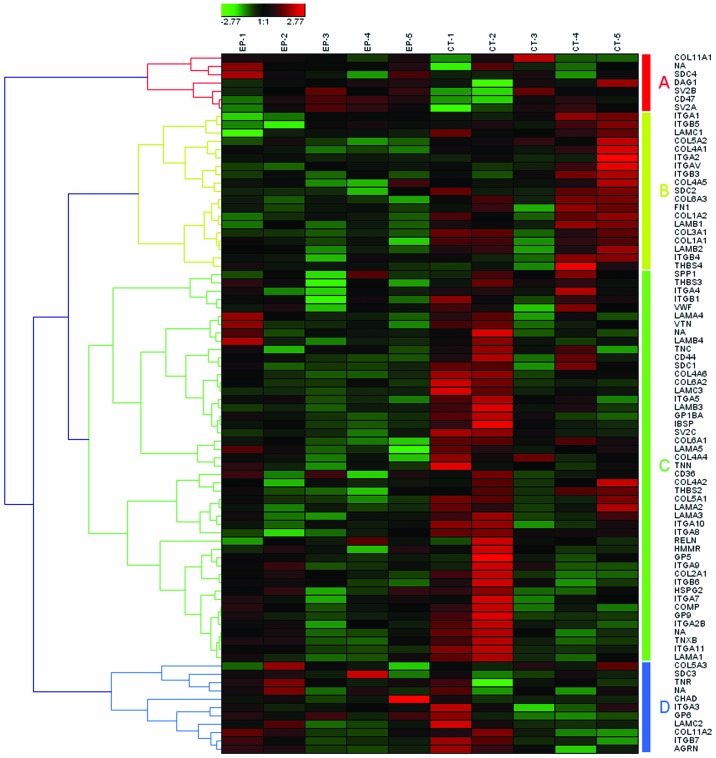
Hierarchical clustering of genes involved in the pathway of extracellular matrix (ECM)-receptor interaction related to epilepsy. In total, there were 84 genes involved in the pathway of ECM-receptor interaction related to epilepsy, which may be clustered into 4 gene set groups (groups A–D) based on hierarchical clustering with Pearson correlation coefficient. Heatmap of 84 genes involved in the pathway of ECM-receptor interaction in 5 epilepsy samples (from EP-1 to EP-5) and 5 control samples (non-epilepsy, from CT-1 to CT-5) is shown. EP, epilepsy; CT, control.

There were 4 and 3 significantly upregulated pathways in the functional class of environmental and genetic information processing, respectively which were associated with epilepsy (Table II). The environmental information processing pathways of ECM-receptor interaction (P=0.004) and CAMs (P=0.020) were related to the functions of signaling molecules and interaction, and the ABC transporter pathway (P=0.030) was related to the function of membrane transport, and the Jak-STAT signaling pathway (P=0.046) was related to the function of signal transduction. The genetic information processing pathways of mismatch repair (P=0.031) and DNA replication (P=0.048) were related to the functions of replication and repair, and the RNA polymerase pathway (P=0.008) was transcription related. Among these, the pathway of ECM-receptor interaction was the most significant in this class, which has also been identified as one of the DEG enriched pathways by Niesen *et al* (10). The ECM is known to regulate important processes in neuronal cell development, activity and growth. The remodeling of the ECM during both development and following injury to the central nervous system has been shown to affect neuronal guidance, synaptic plasticity and their regenerative responses. The functions and potential therapeutic value of several key ECM molecules in epileptogenesis and dementia have been extensively investigated in previous studies (15,16). The 84 genes involved in the pathway of ECM-receptor interaction may also be clustered into 4 gene set groups (Fig. 2, groups A–D).

**Figure 3:**
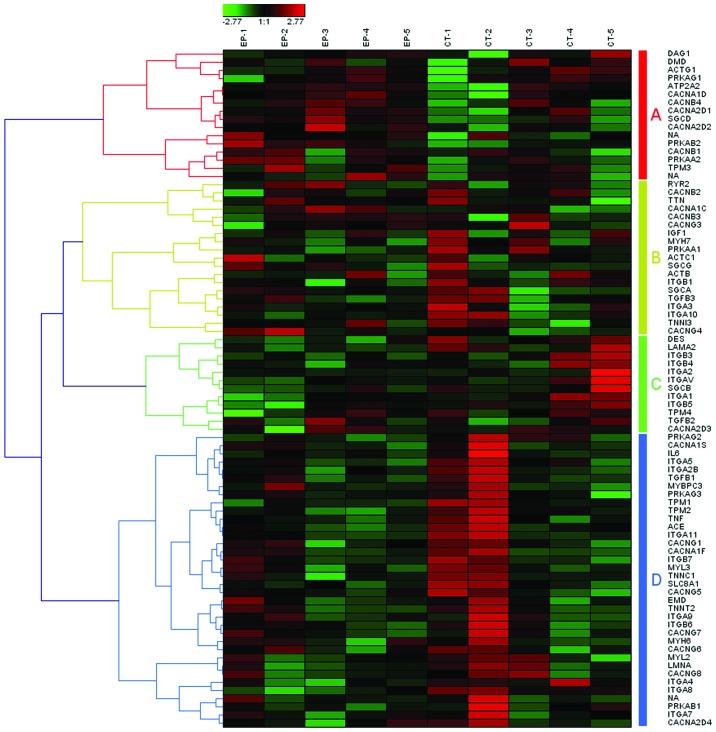
Hierarchical clustering of genes involved in the pathway of hypertrophic cardiomyopathy (HCM) related to epilepsy. In total, there were 83 genes involved in the pathway of HCM related to epilepsy, which may be clustered into 4 gene set groups (groups A–D) based on hierarchical clustering with Pearson correlation coefficient. Heatmap of 83 genes involved in the pathway of HCM in 5 epilepsy samples (from EP-1 to EP-5) and 5 control samples (non-epilepsy, from CT-1 to CT-5) is shown. EP, epilepsy; CT, control.

Twenty-five significantly associated pathways were classified into the functional class of human diseases, including 5 upregulated and 1 downregulated cancer-related pathways, 4 upregulated cardiovascular disease-related pathways, 1 upregulated endocrine and metabolic disease-related pathway, 6 upregulated immune disease-related pathways and 8 upregulated infectious disease-related pathways (Table III). It has been reported that cardiac changes may accompany epilepsy, which may lead to significant seizure-associated cardiac performance decreases (17). These data are consistent with the results from our study which identidied 4 significant cardiovascular disease-related pathways associated with epilepsy. Among these, the pathway of hypertrophic cardiomyopathy (HCM) was one of the most significant cardiovascular disease-related pathways (P=0.004). The association between these 2 diseases has been revealed in a clinical case report (18). In total, there were 83 genes involved in the pathway of HCM associated with epilepsy, which may be clustered into 4 gene set groups (Fig. 3, groups A–D).

**Figure 4:**
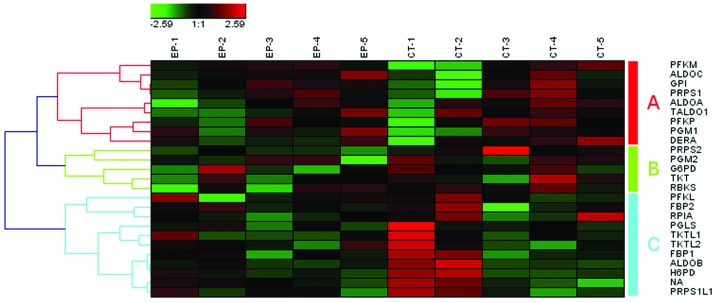
Hierarchical clustering of genes involved in pentose phosphate pathway related to epilepsy. In total, there were 25 genes involved in the pentose phosphate pathway related to epilepsy, which may be clustered into 3 gene set groups (groups A–C) based on hierarchical clustering with Pearson correlation coefficient from group of A to C. Heatmap of 25 genes involved in the pentose phosphate pathway in 5 epilepsy samples (from EP-1 to EP-5) and 5 control samples (non-epilepsy, from CT-1 to CT-5) is shown. EP, epilepsy; CT, control.

In the functional class of metabolism, there were 14 significantly upregulated pathways associated with epilepsy (Table IV). These were involved in 6 different types of metabolism, including amino acid metabolism, carbohydrate metabolism, glycan biosynthesis and metabolism, lipid metabolism, metabolism of co-factors and vitamins, as well as nucleotide metabolism. Among these, the pentose phosphate pathway was one of the most significant pathways (P=0.007), which was classified into the functional group of carbohydrate metabolism. A variety of observations suggested that decreasing glycolysis and increasing the levels of reduced glutathione, generated by the metabolism of glucose through the pentose phosphate pathway, may have an anticonvulsant effect. Fructose-1,6-bisphosphate (F1,6BP) may have anticonvulsant activity in models of acute seizures in adult rats, which shifts the metabolism of glucose from glycolysis to the pentose phosphate pathway (19). The total of 25 genes involved in the pentose phosphate pathway may be clustered into 3 gene set groups (Fig. 4, groups A–C).

**Figure 5:**
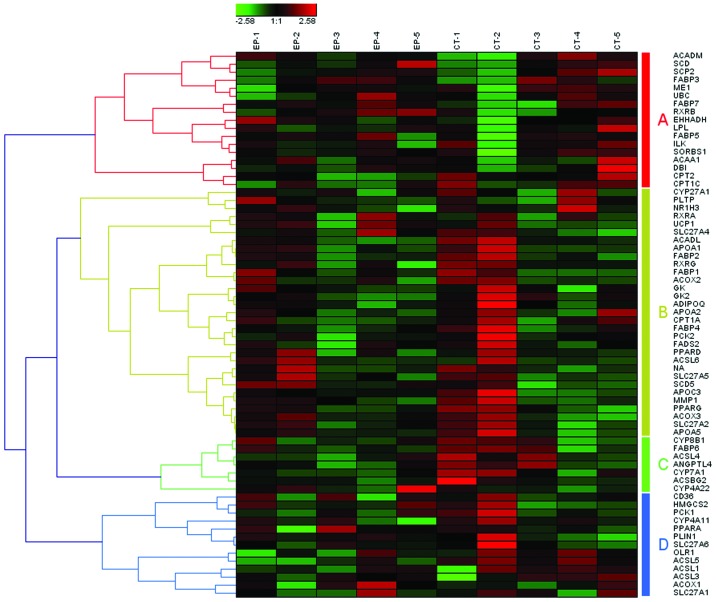
Hierarchical clustering of genes involved in peroxisome proliferato-activated receptor (PPAR) signaling pathway related to epilepsy. In total, there were 68 genes involved in the PPAR signaling pathway related to epilepsy, which may be clustered into 4 gene set groups (groups A–C) based on hierarchical clustering with Pearson correlation coefficient. Heatmap of 68 genes involved in the pentose phosphate pathway in 5 epilepsy samples (from EP-1 to EP-5) and 5 control samples (non-epilepsy, from CT-1 to CT-5) is shown. EP, epilepsy; CT, control.

In the last functional class of organismal systems, there were 2 significantly downregulated and 15 significantly upregulated pathways associated with epilepsy (Table V). These were involved in development, the nervous system, the endocrine system, as well as the immune system. Among these, the peroxisome proliferator-activated receptor (PPAR) signaling pathway was one of the most associated pathways (P=0.045), which was classified into the functional group of endocrine system. PPAR-γ/mitochondrial uncoupling protein 2 signaling has been reported to protect neurons against seizure-induced neuronal cell death in the hippocampus following experimental status epilepticus (20). The activation of the mammalian target of rapamycin or PPAR-γ pathways has been considered as one of the pre-clinical models for the anti-epileptogenic activity of a diverse range of potential therapies (21). Furthermore, there were 68 genes in total which were involved in the PPAR signaling pathway associated with epilepsy, which may be clustered into 4 gene set groups (Fig. 5, groups A–D).

### Candidate TF selection related to epilepsy

To predict the TFs potentially involved in the regulation of epilepsy, we performed the analysis of TFBSs and the prediction of TFs using the significant genes in each identified pathway. Based n the cut-off value of TF importance, we identified the candidate TFs related to epilepsy with potential target genes which were co-regulated in each of the 72 pathways identified. The details are available upon request.

Among these, the TF of PPAR-γ appeared in several pathways, including fatty acid metabolism, pyrimidine metabolism, the PPAR signaling pathway, lysosome, amoebiasis and melanoma (data available upon request). PPARs belong to the nuclear hormone receptor superfamily. They play critical physiological roles as lipid sensors and regulators of lipid metabolism and are activated by fatty acids (22). Previous studies have shown that PPAR-γ has anti-inflammatory effects in seizure animal models (20,23). The anticonvulsant effects of acute pioglitazone on pentylenetetrazol (PTZ)-induced seizures in mice have been demonstrated to be mediated through the PPAR-γ receptor-mediated pathway (24). One of the PPAR-γ agonists, rosiglitazone, has been proven to protect the central nervous system from oxidative damage in epileptic rats, which may be a potential neuroprotective agent for epilepsy (25). Our data also support the idea that PPAR-γ may be one of the essential targets for the management of epilepsy due to brain tumors.

Another essential TF of the phosphatidylethanolamine-binding protein (PEBP) was identified in 3 of 4 downregulated pathways, including the GnRH signaling pathway, gap junction and long-term depression pathways. PEBP is alternatively named Raf-1 kinase inhibitor protein, the precursor of the hippocampal cholinergic neurostimulating peptide (HCNP) corresponding to its natural N-terminal fragment, which has been previously described to be released by hippocampal neurons (26–28). The crystal structure of human PEBP (hPEBP) suggests that the ligand-binding site may accommodate the phosphate head groups of membrane lipids, therefore allowing the protein to adhere to the inner leaf of bilipid membranes where it would be ideally positioned to relay signals from the membrane to the cytoplasm (29). With the previous evidence of calpain dysregulation in Alzheimer’s disease (AD), PEBP has been confirmed as a novel *in vitro* and *in situ c*alpain substrate using an *in vitro* proteomics approach or serial analysis of gene expression (SAGE) (30,31). In particular, during brain development, the N-terminal part of mammalian PEBP has been reported to be specifically cleaved and the resulting 11 amino acid peptides may stimulate the growth and activity of acetylcholinergic neurons (32).

## Figures and Tables

**Table I:** Significant pathways associated with epilepsy in the functional class of cellular processes.

Pathways	Map B	P-value	No. of genes	No. of TFs
04510: Focal adhesion	Cell communication	0.009	199	33
04540^a^: Gap junction	Cell communication	0.034	86	27
04210: Apoptosis	Cell growth and death	<0.001	87	28
04115: p53 signaling pathway	Cell growth and death	0.018	68	31
04110: Cell cycle	Cell growth and death	0.035	122	37
04810: Regulation of actin cytoskeleton	Cell motility	0.048	211	37
04146: Peroxisome	Transport and catabolism	<0.001	78	29
04142: Lysosome	Transport and catabolism	0.014	111	26
04145: Phagosome	Transport and catabolism	0.031	141	29

a Represents the significantly downregulated pathways associated with epilepsy.

TFs, transcription factors.

**Table II:** Significant pathways associated with epilepsy in the functional class of environmental and genetic information processing.

Pathways	Map B	P-value	No. of genes	No. of TFs
04512: ECM-receptor interaction	Signaling molecules and interaction	0.004	84	28
04514: Cell adhesion molecules (CAMs)	Signaling molecules and interaction	0.020	128	41
02010: ABC transporters	Membrane transport	0.030	43	17
04630: Jak-STAT signaling pathway	Signal transduction	0.046	153	33
03020: RNA polymerase	Transcription	0.008	27	20
03430: Mismatch repair	Replication and repair	0.031	21	20
03030: DNA replication	Replication and repair	0.048	35	11

TFs, transcription factors; ECM, extracellular matrix.

**Table III:** Significant pathways associated with epilepsy in the functional class of human diseases.

Pathways	Map B	P-value	No. of genes	No. of TFs
05222: Small cell lung cancer	Cancers	0.008	85	38
05213^a^: Endometrial cancer	Cancers	0.008	52	30
05212: Pancreatic cancer	Cancers	0.011	69	40
05218: Melanoma	Cancers	0.012	71	40
05200: Pathways in cancer	Cancers	0.022	323	30
05219: Bladder cancer	Cancers	0.023	41	16
05410: Hypertrophic cardiomyopathy (HCM)	Cardiovascular diseases	0.004	83	37
05412: Arrhythmogenic right ventricular cardiomyopathy (ARVC)	Cardiovascular diseases	0.004	74	33
05416: Viral myocarditis	Cardiovascular diseases	0.034	64	21
05414: Dilated cardiomyopathy	Cardiovascular diseases	0.049	90	37
04940: Type I diabetes mellitus	Endocrine and metabolic diseases	0.013	39	24
05323: Rheumatoid arthritis	Immune diseases	0.004	83	19
05322: Systemic lupus erythematosus	Immune diseases	0.013	82	24
05320: Autoimmune thyroid disease	Immune diseases	0.013	47	20
05330: Allograft rejection	Immune diseases	0.013	33	14
05332: Graft-versus-host disease	Immune diseases	0.013	33	21
05310: Asthma	Immune diseases	0.020	26	27
05100: Bacterial invasion of epithelial cells	Infectious diseases: Bacterial	0.009	70	36
05150: Staphylococcus aureus infection	Infectious diseases: Bacterial	0.013	48	19
05146: Amoebiasis	Infectious diseases: Parasitic	<0.001	106	25
05145: Toxoplasmosis	Infectious diseases: Parasitic	0.008	120	35
05140: Leishmaniasis	Infectious diseases: Parasitic	0.008	66	25
05144: Malaria	Infectious diseases: Parasitic	0.024	48	28
05142: Chagas disease (American trypanosomiasis)	Infectious diseases: Parasitic	0.036	101	41
05160: Hepatitis C	Infectious diseases: Viral	0.018	131	38

a Represents the significantly downregulated pathways associated with epilepsy.

TFs, transcription factors.

**Table IV:** Significant pathways associated with epilepsy in the functional class of metabolism.

Pathways	Map B	P-value	No. of genes	No. of TFs
00360: Phenylalanine metabolism	Amino acid metabolism	0.031	17	26
00380: Tryptophan metabolism	Amino acid metabolism	0.039	42	21
00030: Pentose phosphate pathway	Carbohydrate metabolism	0.007	25	39
00520: Amino sugar and nucleotide sugar metabolism	Carbohydrate metabolism	0.018	46	29
00010: Glycolysis/gluconeogenesis	Carbohydrate metabolism	0.024	63	25
00511: Other glycan degradation	Glycan biosynthesis and metabolism	0.014	16	22
00604: Glycosphingolipid biosynthesis - ganglio series	Glycan biosynthesis and metabolism	0.043	15	26
00071: Fatty acid metabolism	Lipid metabolism	0.021	42	22
00100: Steroid biosynthesis	Lipid metabolism	0.032	18	21
00860: Porphyrin and chlorophyll metabolism	Metabolism of cofactors and vitamins	0.009	30	23
00770: Pantothenate and CoA biosynthesis	Metabolism of cofactors and vitamins	0.013	16	20
00480: Glutathione metabolism	Metabolism of other amino acids	0.022	47	19
00230: Purine metabolism	Nucleotide metabolism	<0.001	155	26
00240: Pyrimidine metabolism	Nucleotide metabolism	0.029	92	43

TFs, transcription factors.

**Table V:** Significant pathways associated with epilepsy in the functional class of organismal systems.

Pathways	Map B	P-value	No. of genes	No. of TFs
04380: Osteoclast differentiation	Development	0.004	125	45
04730^a^: Long-term depression	Nervous system	0.004	65	46
04920: Adipocytokine signaling pathway	Endocrine system	0.027	67	31
04912^a^: GnRH signaling pathway	Endocrine system	0.029	94	25
03320: PPAR signaling pathway	Endocrine system	0.045	68	21
04650: Natural killer cell mediated cytotoxicity	Immune system	0.004	125	31
04670: Leukocyte transendothelial migration	Immune system	0.009	113	46
04610: Complement and coagulation cascades	Immune system	0.013	67	25
04672: Intestinal immune network for IgA production	Immune system	0.013	44	30
04640: Hematopoietic cell lineage	Immune system	0.014	84	31
04612: Antigen processing and presentation	Immune system	0.017	63	21
04666: Fc gamma R-mediated phagocytosis	Immune system	0.018	90	31
04620: Toll-like receptor signaling pathway	Immune system	0.024	99	30
04662: B cell receptor signaling pathway	Immune system	0.028	74	43
04621: NOD-like receptor signaling pathway	Immune system	0.032	58	20
04623: Cytosolic DNA-sensing pathway	Immune system	0.033	53	17
04660: T cell receptor signaling pathway	Immune system	0.048	108	35

a Represents the significantly downregulated pathways associated with epilepsy.

TFs, transcription factors; PPAR, peroxisome proliferator-activated receptor.
